# A high-dimensional platform for observing neutrophil–parasite interactions

**DOI:** 10.1128/spectrum.00472-24

**Published:** 2024-06-18

**Authors:** Brandon A. Thompson, Julio Revilla, Savannah Brovero, Stacey L. Burgess

**Affiliations:** 1 Division of Infectious Diseases and International Health, Department of Internal Medicine, University of Virginia School of Medicine, Charlottesville, Virginia, USA; Hubei University of Medicine, Shiyan, China

**Keywords:** spectral flow, amebiasis, *Entamoeba*, neutrophils, parasitology, gut microbiome

## Abstract

Diarrheal diseases with infectious etiology remain a major cause of death globally, particularly in low-income countries. *Entamoeba histolytica* is a pathogenic protozoan parasite that is the causative agent of amebiasis. Amebiasis has a wide presentation in clinical severity with many factors, including the bacterial microbiota, contributing to this variation. The innate immune response also plays a critical role in regulating the severity of *E. histolytica* infection, with neutrophils reported to have a protective role. Despite this, the precise mechanism of how neutrophils mediate amebic killing is poorly understood. Thus, modern platforms that allow for inquiry of granulocyte–ameba interactions will increase our understanding of this disease. Herein, we describe an assay for neutrophil killing of *E. histolytica* by utilizing high-dimensional spectral flow cytometry. Neutrophils were isolated from wild-type 5-week-old C57BL/6 mice and co-cultured with *E. histolytica* at various multiplicity of infections (MOIs). After co-culture, neutrophils and *E. histolytica* were stained for spectral flow cytometry. Cell populations were identified using surface markers and fluorescence minus one (FMO) controls. We have previously shown that animals colonized with a component of the human microbiota, *Clostridium scindens*, were protected from *E. histolytica*. This protection was associated with elevated neutrophil count. Here, we explored amebic killing capacity and observed that neutrophils from animals with *C. scindens* possessed heightened amebic killing compared with controls. Thus, this study establishes a novel platform that can provide an in-depth analysis of granulocyte–parasite interactions in various contexts, including during alteration of the intestinal microbiota.

**IMPORTANCE:**

The tools for studying host immune cell*–E. histolytica* interactions are limited. Factors, such as parasite heterogeneity, infectivity, and difficulties with culture systems and animal models, make interrogation of these interactions challenging. Thus, *Entamoeba* researchers can benefit from next-generation models that allow for the analysis of both host and parasite cells. Here, we demonstrate the use of a novel platform that allows for the determination of parasite–host cell interactions and customizable high-dimensional phenotyping of both populations. Indeed, spectral flow cytometry can approach >40 markers on a single panel and can be paired with custom-developed parasite antibodies that can be conjugated to fluorochromes via commercially available kits. This platform affords researchers the capability to test highly precise hypotheses regarding host–parasite interactions.

## INTRODUCTION

Diarrheal diseases remain a major cause of mortality globally, particularly in children <5 years of age in low-income countries (1, 2). *Entamoeba histolytica* is a pathogenic protozoan parasite that is the causative agent of amebic diarrhea and can cause intestinal colitis and extraintestinal complications, including amebic liver abscess, in severe cases (3, 4). After ingestion, trophozoites colonize and invade intestinal tissue by adhering to the colonic mucin layer via the expression of the galactose and *N*-acetyl-D-galactosamine specific lectin (Gal/GalNAc lectin) (5, 6). During infection, *E. histolytica* can cause extensive tissue damage via virulence factors that kill intestinal epithelial cells and degrade the colonic mucin layer, including cysteine proteases and amebapores (7–10). Currently, there is no vaccine for *E. histolytica* infection and the primary treatment, metronidazole, while effective at clearing trophozoites, has shown limited efficacy against cysts in the colonic lumen (11). Additional treatments are required to completely resolve the infection, such as paromomycin or iodoquinol, which can result in patient non-compliance due to treatment complexity and cost (12). Thus, there is a need to better understand symptomatic *E. histolytica* infection and develop therapeutics to combat the disease. Recent studies have highlighted the importance of the innate immune response, particularly neutrophils, in mediating protection from *E. histolytica* infection.

Neutrophils are one of the most abundant circulating leukocytes composing 30%/70% of all circulating white blood cells in mice/humans, respectively (13, 14). Neutrophils are critically important in most infections and are abundant in *E. histolytica* lesions, whereas other innate immune cells, such as macrophages, are not, highlighting their potential importance during amebic infections (15). Previous work established an intimate relationship between neutrophils and *E. histolytica*. Indeed, *E. histolytica* infection is much more severe in neutropenic animals compared with control animals (16, 17). Similarly, neutrophil chemoattractant interleukin-8 (IL-8) is significantly elevated during *E. histolytica* infection (18, 19). Antibiotic induced dysbiosis in *E. histolytica* infection-resistant C57BL/6 mice rendered them susceptible to infection by downregulating neutrophil expression of CXCR2. This likely decreased intestinal neutrophil recruitment by diminishing their chemotactic capacity and function in the intestinal environment (20). Similarly, our group demonstrated that animals colonized with human commensal bacteria*, Clostridium scindens*, are protected from *E. histolytica* infection. This protection is associated with higher numbers of intestinal neutrophils (21). Although these studies demonstrate the role of neutrophils during *E. histolytica* infection, more comprehensive systems are needed to fully understand how neutrophils mediate amebic clearance.

*In vivo* murine models allow for the study of *E. histolytica* infection in the context of a complete vertebrate immune system. However, notable issues exist, such as low infectivity and limited ability to directly study immune cell*–E. histolytica* interactions (22, 23). Moreover, during *in vivo* experimentation, it can be difficult to decouple the effects of elevated neutrophil numbers from heightened neutrophil antimicrobial function. Researchers have developed neutrophil*–E. histolytica* co-culture assays with the purpose of studying short-term interactions *ex vivo*. This allows for the study of neutrophil killing capacity across treatments while holding cell numbers constant. These methodologies have been used for the study of neutrophil extracellular trap release (NETosis), an important method that neutrophils use to kill ameba, and more recently, the release of extracellular vesicles from neutrophils (24–27). These assays can also be utilized for targeted quantification of extracellular proteins, and fluorescence microscopy has been recently used to analyze markers of interest on neutrophils or *E. histolytica*. However, fluorescence microscopy is inherently limited in the number of cellular parameters that can be analyzed in tandem and thus would not be suitable for researchers desiring a multi-dimensional approach. Importantly, recent single-cell RNAseq studies have demonstrated that neutrophils are more heterogenous than previously thought (28, 29). Thus, there exists an urgent need for *ex vivo* approaches allowing for multi-parameter and cell-based analysis of *E. histolytica*–neutrophil interactions.

Herein, we describe a novel platform that builds upon previously existing assays while utilizing high-dimensional spectral flow cytometry as a readout. This allows for in-depth interrogation of cellular and *E. histolytica* markers following co-culture. This assay can be expanded, depending on the researchers’ needs, up to 40+ markers. We demonstrated the identification of both *E. histolytica* and murine neutrophils after a 1-h co-culture and could observe both amebic and neutrophil cell death. We then utilized this platform to test how targeted alteration of the microbiota might alter neutrophil killing capacity. We observed that neutrophils from mice with *C. scindens* have a higher capacity to kill *E. histolytica,* independent of cell number, affording us greater clarity regarding how *C. scindens* protects from amebic infection. Thus, our assay provides researchers with a platform to ask targeted questions regarding *E. histolytica*–neutrophil interactions via a high-dimensional analysis that can be both hypothesis testing and generating.

## RESULTS

### Development of a spectral cytometry-based parasite killing assay

**Fig 1:**
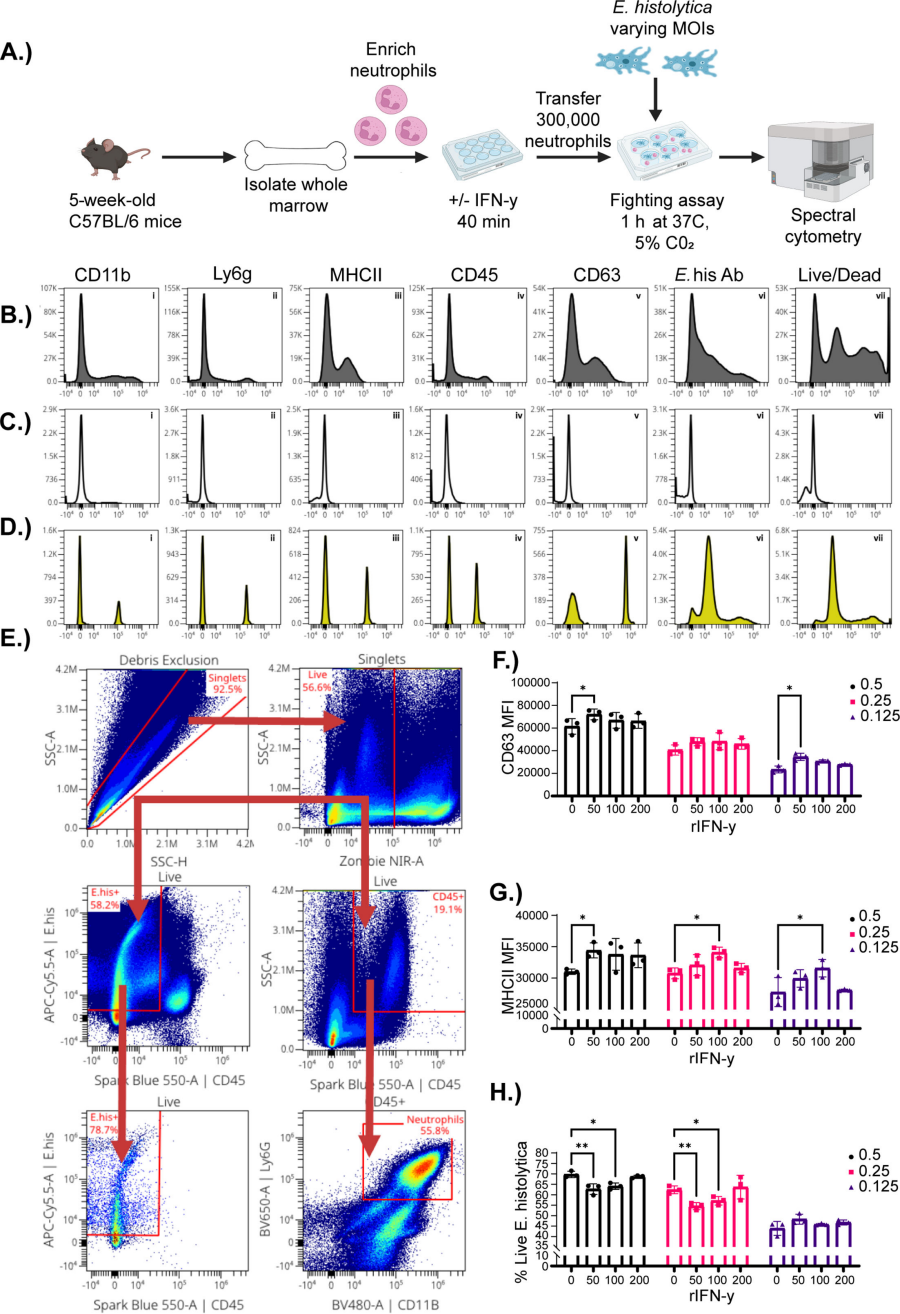
Creation of a spectral cytometry-based killing assay for *Entamoeba histolytica*. Graphical representation of methods. Briefly, whole marrow was taken from wild-type C57BL/6 mice, and neutrophils were enriched by utilizing magnetically activated cell sorting (MACS)-based isolation. Neutrophils were then cultured with 0, 50, 100, or 200 U/mL of recombinant murine IFN-γ for 40 min at 37°C and 5% CO_2_. Neutrophils were transferred to plates with *E. histolytica* at an MOI of 0.5, 0.25, or 0.125 and allowed to interact for 1 h. Neutrophils and ameba were then removed from co-culture and stained for spectral flow cytometry. Flow analysis was performed, and histograms were created to determine the efficacy of staining of antibodies used in (**B**) fully stained neutrophils and *E. histolytica*, (**C**) FMOs, and (**D**) single stains. (**E**) Gating strategy to identify neutrophils and *E. histolytica*; arrows indicate the path taken for stepwise determination of cell populations (neutrophils: live, CD45^+^, CD11b^+^, and Ly6G^+^; *E. histolytica*: live, CD45– and *E. histolytica*^+^). The mean fluorescent intensity (MFI) of neutrophil activation markers (**F**)CD63, (**G**)MHCII, and (**H**) percentage of live *E. histolytica* was analyzed across IFN-γ treatment concentration and MOI. Data were analyzed using two-way analysis of variance (ANOVA) and Dunnet’s post-hoc test *P < 0.05; **P < 0.01.

Innate immune cells, particularly neutrophils, play a significant role in protection from *E. histolytica* infection. However, only a few tools investigate neutrophil*–E. histolytica* interactions. Although researchers have utilized neutrophil*–E. histolytica* co-culture assays to address questions of short-term interactions, these existing platforms do not allow for in-depth phenotyping. Thus, we designed a novel methodology that would utilize existing knowledge in the field and pair it with the high-dimensional phenotyping capacity of flow cytometry (Fig. 1A). We began by ensuring that we could identify both neutrophils and *E. histolytica* by analyzing fully stained samples (Fig. 1B), fluorescence minus one (FMO) controls (Fig. 1C) and single-stained samples (Fig. 1D). Using the FMO controls, we designed a gating strategy to identify both the neutrophils and *E. histolytica* (Fig. 1E). Neutrophils were identified as CD45^+^, CD11b^+^, and ly6G^+^, and *E. histolytica* was identified as CD45^−^ and positive for an *E. histolytica* monoclonal antibody conjugated to APC-Cy5.5 (Fig. 1E). The neutrophil expression of CD63 and MHCII was analyzed because interferon (IFN)-γ treatment has previously been associated with their upregulation and an increase in amebicidal activity (30–32). We observed that expression of CD63 and MHCII was dependent on MOI and IFN-γ concentration in our assay (Fig. 1F and G). Next, we explored the most appropriate MOI for the assay by analyzing parasite death during co-culture. We observed a higher rate of amebic death in cultures at an MOI of 0.5 and 0.25 when previously treated with 50 and 100 U/mL of IFN-γ (Fig. 1H). Thus, we utilized these parameters to perform the assay and begin to test hypotheses about alteration of the microbiota and neutrophil killing capacity.

### Neutrophils from *C. scindens*-colonized mice are more potent at killing ameba *in vitro*

**Fig 2:**
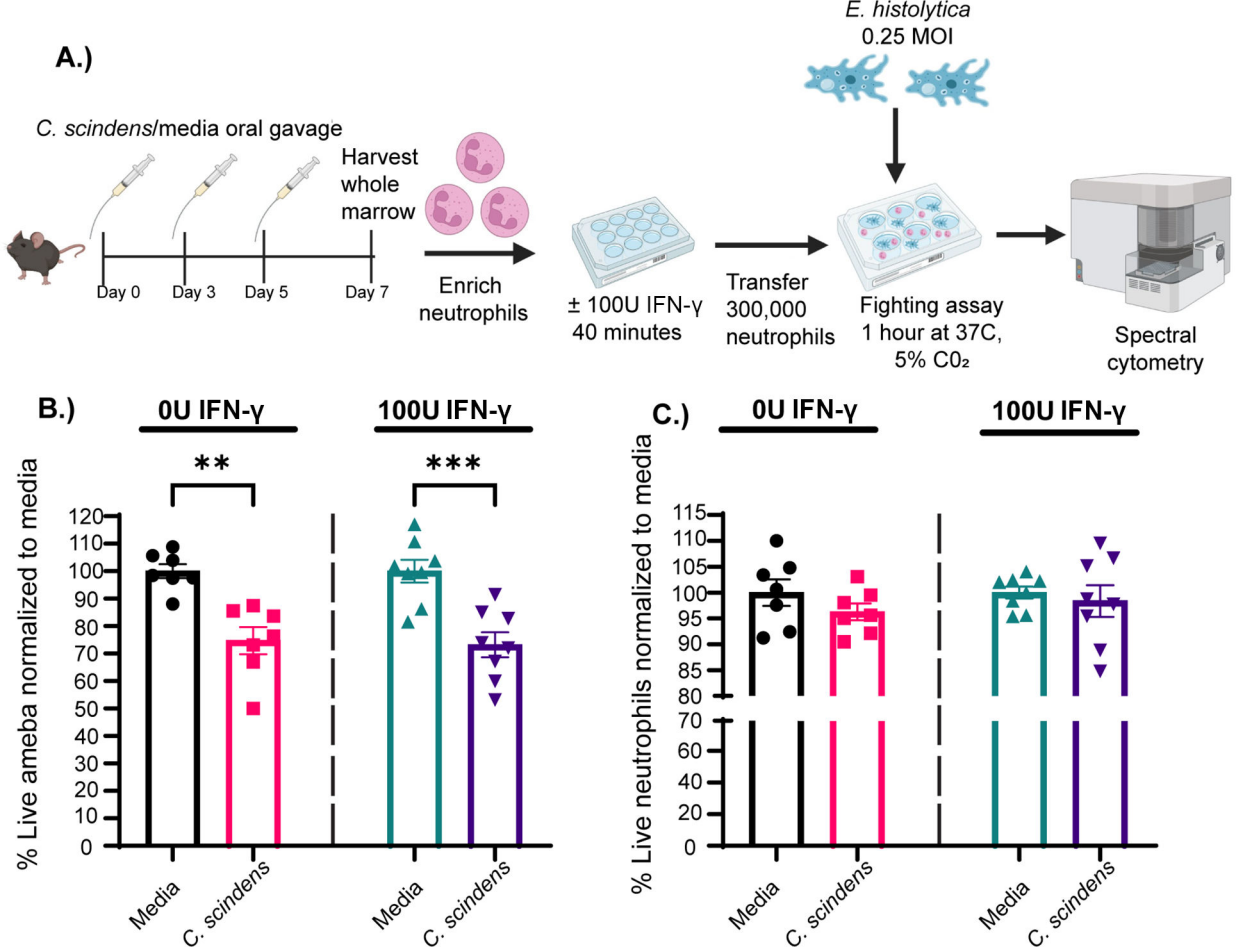
Neutrophils from *Clostridium scindens*-colonized mice have heightened amebicidal activity. (**A**) Graphical representation of methods. The 5-week-old C57BL/6 mice were administered thrice with human commensal bacteria *C. scindens* over 7 days. Whole marrow was harvested at day seven, and bone marrow neutrophils were utilized in our ameba fighting assay described in Fig. 1. (**B**) Percentage of live *E. histolytica* and (**C**) neutrophils treated with 0 U/mL or 100 U/mL of IFN-γ normalized to media control averages. Data were analyzed with a one-way analysis of variance (ANOVA) with Tukey’s post-hoc test. *P < 0.05, **P < 0.01, ***P < 0.001.

We have demonstrated that *C. scindens* colonization is associated with an influx of intestinal neutrophils and protection from *E. histolytica* in a murine model of amebic infection (33). However, it remained unknown whether this protection was mediated solely by an increase in neutrophil numbers or if functionally different neutrophils are generated during *C. scindens* colonization than under homeostatic conditions. We utilized our newly developed neutrophil*–E. histolytica* co-culture assay to test the hypothesis that neutrophils from *C. scindens*-colonized mice might differentially kill ameba. This assay allowed us to test this idea by holding neutrophil numbers constant while exploring neutrophil amebic killing capacity. Neutrophils were isolated from both *C. scindens*-colonized and uncolonized mice and placed into our neutrophil*–E. histolytica* co-culture assay to test if neutrophils from mice with *C. scindens* would differentially kill ameba (Fig. 2A). Interestingly, we observed higher rate of amebic death when cultured with neutrophils from animals colonized with *C. scindens* (Fig. 2B). We also showed that neutrophil death was unchanged in the assay between colonized and uncolonized controls (Fig. 2C). These data suggest that *C. scindens* colonization elevates the amebicidal capacity of neutrophils in a manner that does not increase neutrophilic death, such as NETosis (34). However, future studies will examine the precise mechanism of this increased killing and the possibility of neutrophil heterogeneity with alteration of the microbiota. Overall, these observations strengthen the validity of our platform as a method of interrogating granulocyte*–E. histolytica* interactions as they relate to infectious severity and host environmental factors, such as the microbiota.

## DISCUSSION

The key development of this work is the establishment of a platform to analyze granulocyte–ameba interactions that allows for high-dimensional, whole-cell-based phenotyping of both neutrophils and ameba. Previously, researchers utilizing neutrophil*–E. histolytica* co-culture have discovered the existence of a reciprocal relationship between neutrophils and *E. histolytica*. Indeed, *E. histolytica* can induce neutrophils to undergo NETosis, ultimately killing the trophozoite; however, the ameba can also disrupt NADPH oxidase to inhibit respiratory burst in neutrophils, limiting their killing capacity (27, 35, 36). Considering the importance of neutrophils in mediating protection from *E. histolytica* infection, we posit that the platform developed here could be used by researchers to better understand these interactions and others. Spectral flow cytometry affords researchers the unique ability to expand the scope of their experiments to 40+ markers via the development of multi-parameter highly targeted panels (37). Moreover, by using commercially available fluorochrome conjugation kits, in-lab-produced antibodies can be readily prepared for use in flow cytometry, allowing identification and quantification, via mean fluorescence intensity (MFI), of *E. histolytica*/neutrophil proteins of interest. The workflow laid out here could also be adapted to examine other important granulocytes during *E. histolytica* infection, such as eosinophils (38, 39). Researchers could utilize this platform to better understand *E. histolytica*–neutrophil interactions while allowing for in-depth interrogation of host cell anti-microbial activity and amebic virulence factors and effectors within the same workflow.

Approximately 90% of *E. histolytica* infections are asymptomatic, and little is known about why some individuals are protected and others experience severe disease (40, 41). Interestingly, previous studies of global human cohorts have suggested a relationship between intestinal microbiome composition and *E. histolytica* infection severity in humans (42, 43). A recent study comparing asymptomatic vs symptomatic *E. histolytica* infections in a Japanese cohort found that symptomatic invasive cases were associated with a higher abundance of Streptococcacea, and a lower abundance of Ruminococcacaea, Coriobacteriacaea, and Clostridiaceae (44). It has been appreciated that the microbiome and its metabolites can have profound effects on mature neutrophil function and granulopoiesis (20, 45–49). These studies suggest that the microbiome partially alters amebic disease severity through alterations in neutrophil function/generation. Utilizing our co-culture assay, we demonstrated higher amebic death in cultures containing neutrophils from animals colonized with *C. scindens*. These data not only validate our platform as a viable way to study granulocyte*–E. histolytica* interactions but also suggest that *C. scindens* colonization directly alters the amebic killing capacity of neutrophils.

It is also important to note that *C. scindens* expresses enzymes capable of producing potent immunostimulatory metabolites known as secondary bile acids, such as deoxycholic acid (DCA) and lithocholic acid (LCA), and that colonization elevates serum concentrations of DCA and LCA (50–52). Interestingly, work in our laboratory has suggested that secondary bile acids are important in mediating protection from *E. histolytica* with both children and mice with elevated serum secondary bile acids being protected from amebic infection (33). Administration of secondary bile acids was also sufficient to elevate intestinal neutrophil numbers during infection and increase neutrophil production from hematopoietic progenitors in the absence of *C. scindens* (53). Importantly, elevated neutrophil count is only observed during infectious challenge and not at baseline, suggesting that secondary bile acids prime the immune system to respond to future insults (21). Moreover, short-term treatments of hematopoietic progenitor cells *in vitro* and bone marrow transplant studies demonstrate that transient elevations in secondary bile acids can result in lasting changes to myelopoiesis, which could help guide their use as therapeutics by modulating tissue damage by bile overproduction (21, 53). Future studies utilizing this backbone platform will allow for the examination of the contribution of secondary bile acids and other microbial metabolites to neutrophil killing of ameba. Overall, this assay provides the parasitology research community a platform to better understand neutrophil*–E. histolytica* interactions in a high-dimensional manner and may facilitate more in-depth understanding of factors contributing to asymptomatic vs symptomatic infection during amebiasis.

## MATERIALS AND METHODS

### Mice

The 5-week-old male C57BL/6 mice (Jackson Laboratories) were housed in a specific pathogen-free facility and provided with autoclaved food and water *ad libitum*. Sentinel mice were used in the facility to ensure that mice were free of common murine pathogens. All procedures were approved by the institutional animal care and use committee of the University of Virginia.

### *E. histolytica* culture and preparation

A xenic culture of animal-passaged *E. histolytica* (laboratory strain HM1:IMSS) trophozoites was maintained from cecal contents of infected mice in complete trypsin-yeast-iron (TYI) medium supplemented with 10,000 U/mL of penicillin and streptomycin (Gibco), Diamond Vitamin mixture (Sigma-Aldrich) and 5% heat-inactivated adult bovine serum (Sigma-Aldrich). One week before the neutrophil co-culture experiments, *E. histolytica* was passaged into T25 culture flasks, grown to confluence, and subsequently passaged into larger T75s to ensure enough parasites for the assay.

### Bone marrow isolation and neutrophil enrichment/priming

Whole bone marrow was isolated as previously described (33, 53) Briefly, tibias and fibulas from 5-week-old male C57BL/6 mice were cut at one end to expose the bone marrow and were placed into a 0.5 mL micro-centrifuge tube with a hole in the bottom, marrow side down, nested in a 1.5 mL Eppendorf tube. Subsequently, 100 μL of complete media (RPMI + 10% FBS and 5.7 mM L-cysteine) was added to each set of nested tubes and centrifuged at max speed (14,800 RPM/ 16,162*×g*) on a tabletop micro-centrifuge (Microfuge 16, Beckman Coulter) to isolate the bone marrow. Before neutrophil enrichment, whole marrow was counted at a 1:10 dilution using trypan blue exclusion on an automatic hemocytometer to ensure appropriate cell numbers for enrichment (TC-20; Bio-Rad). Whole marrow was centrifuged at 800×*g* for 7 min, and complete media were decanted and replaced with 200 μL of magnetic-associated cell sorting (MACS) buffer (Auto Macs Rinsing Solution [Miltenyi Biotec] + 5% BSA [Miltenyi Biotec]). Neutrophils were enriched via MACS utilizing a neutrophil isolation kit (Miltenyi Biotec) and LS columns (Miltenyi Biotec) according to manufacturer’s instructions. Sorted neutrophils were centrifuged at 800×*g* for 7 min, MACS buffer was removed and replaced with 1 mL of complete media, and neutrophils were counted using an automatic hemocytometer (TC-20; Bio-Rad). Subsequently, 300,000 neutrophils were transferred to deep well plates (Axygen), centrifuged at 800×*g* for 7 min. Media were decanted, and cells were resuspended gently with 1 mL of complete media supplemented with 0, 50, 100, or 200 U/mL of recombinant murine IFN-γ (Biolegend) and placed into a 37°C incubator for 40 min to prime the neutrophils before the *E. histolytica* co-culture.

### Neutrophil*–E. histolytica* co-culture

On the morning of the assay before bone marrow isolation, *E. histolytica* culture flasks were placed upright to allow dead/dying non-adherent parasites to aggregate at the bottom. While neutrophils were priming in the incubator *E. histolytica* trophozoites were removed from culture flasks by first removing most of the media containing dead/dying parasites. T75 culture flasks were then rapidly tapped horizontally onto the biosafety cabinet for approximately 1 min to dislodge living cells from the flask wall. Parasites were then transferred to 50 mL falcon tubes (Fisher Scientific) and centrifuged at 900×*g* for 6 min. Media were removed and replaced with 25 mL of complete media, and parasites were counted using trypan blue exclusion on an automatic hemocytometer (TC-20; Bio-Rad). Neutrophils were then removed from the incubator, centrifuged at 800×*g* for 7 min, media were decanted, cells were washed with complete media, centrifuged again, and finally resuspended with 1 mL of complete media and transferred to a six-well plate with *E. histolytica* at multiplicity of infections (MOIs) of 0.125, 0.25, and 0.5 for a total of 2 mL of complete media. Then, 500 μL of sterile RNAase free water (Invitrogen) was added to the space between the wells to provide humidity, and the plates were placed into a 37°C 5% CO_2_ incubator for 1 h. Media were then removed, being careful to not disrupt adherent cells, spun down at 800×*g* for 7 min to pellet dead/non-adherent cells, and media were stored at −80°C for later analysis. Adherent cells were then washed with PBS and treated with trypsin–EDTA (0.25%; Thermo Fisher Scientific) until no longer adherent. Trypsin was then removed and placed into the same tube containing the previously centrifuged cells and PBS wash. Then, 700 μL of complete media was added to the cells to stop trypsinization, removed, and placed into the same tube as previous washes. The samples were then centrifuged at 800×*g* for 7 min, media were removed, and cells were resuspended and washed in 1 mL of fluorescence activated cell sorting (FACS) buffer (PBS + 1% FBS), then centrifuged at 800xG for 7 minutes, media was decanted, and cells were resuspended in 350 μL of FACS buffer.

### Spectral flow cytometry preparation and analysis

Before the assay date, a commercially available *E. histolytica* antibody (Thermo Fisher) was conjugated to APC-Cy5.5 by utilizing the lightning link conjugation kit (Abcam). Once cells/parasites were isolated from the co-culture assay, as described above, they were prepped for spectral flow cytometry. Subsequently, 300 μL from each sample was transferred onto a V-bottom plate withholding 50 μL for FMO controls. Neutrophils/parasites were stained with fluorochrome-conjugated antibodies diluted in FACS buffer and placed into IC fixation buffer (eBiosciences) once fully stained. Live cells were analyzed using Live Dead Zombie NIR (Biolegend). Spectral flow experiments were all performed on the Aurora Borealis or the Aurora Northern Lights (Cytek). All gating strategies were developed with FMO controls. Control samples consisting of whole marrow, MACS-sorted neutrophils, and IFN-γ-primed neutrophils not co-cultured with *E. histolytica* were used to ensure that 1.) neutrophils could be accurately and reproducibly identified throughout the assay, and 2.) neutrophils were not lost during manipulations throughout the assay. Similarly, a sample comprised of *E. histolytica* both unstained and single stained with the *E. histolytica* antibody without neutrophils were used to initially identify the parasite via spectral flow cytometry and served as a guideline for analysis. Refer below for a full list of antibodies used (Table 1).

**TABLE 1:** Spectral cytometry flow panel

Marker	Fluorochrome	Supplier	Clone	Cat #
CD11b	BV480	BD Biosciences	M1/70	566117
Ly6g	BV650	Biolegend	1A8	127641
MHCII	BV785	Biolegend	M5/114.15.2	107645
CD45	Spark Blue 550	Biolegend	30-F11	103166
CD63	PE	Thermo Fisher scientific	NVG-2	12–0631-82
Eh Mab	APC-Cy5.5	Eh Mab-Thermo Fisher Scientific APC-Cy5.5 conjugation kit - Abcam	EH34.5	Eh MAB-MA110655 Conjugation kit-ab102855
Live/Dead	Zombie NIR	Biolegend	N/A	423105

*Gating strategies*: Neutrophils/*E. histolytica* were identified as follows: neutrophils: singlets, live, CD45+, CD11b+, and Ly6G+; *E. histolytica*: singlets, live, CD45^−^, *E. histolytica* antibody+

### *C. scindens* colonization via oral gavage

The 5-week-old male C57BL/6 mice (Jackson Laboratories) were colonized with human commensal bacteria *C. scindens* (ATCC 35704) via three oral gavages 1 week before bone marrow harvest and collection as previously described (21). Mice were gavaged with 100 μL of *C. scindens* monoculture at an optical density of 1.1–1.4 at 595 nm or media control (BHI, Anaerobe Systems, AS-872). For a list of all materials used, please refer to the list below (Table 2).

**TABLE 2:** Collection of reagents and materials used during the co-culture assay

Product	Supplier		CAT #	
*Entamoeba histolytica*	Lab grown: Animal passaged		Strain HM1:IMSS	
Lab-made complete trypsin–yeast–iron (TYI) medium. Recipe makes 17.5 L in MilliQ water	Product (g)	Supplier		CAT #
	Biosate Peptone (144)	Fisher Scientific		B11862
	D-(+)-Glucose (48)	Sigma Aldrich		G-8270
	NaCl (9.6)	Fisher Scientific		S640-3
	K_2_HPO_4_ (4.8)	Mallinckrodt		P288-500
	KH_2_PO4 (2.88)	Sigma Aldrich		7100
	L-Cysteine (5.4)	Sigma Aldrich		C-7880
	Ascorbic Acid (0.96)	Sigma Aldrich		A-0278
	Ferric Ammonium Citrate (0.108)	J.T. Baker		1977–01
Pen/Strep	Gibco		15140122	
Diamond vitamin	Sigma-Aldrich		58,980C	
Adult bovine serum	Gemini Bio-Products		100–101	
RPMI medium	Gibco		11875093	
Trypan blue	Sigma-Aldrich		T8154	
Auto MACS rinsing solution	Miltenyi Biotec		130–091-222	
Bovine serum albumin (BSA)	Miltenyi Biotec		130–091-376	
Neutrophil Isolation Kit	Miltenyi Biotec		130–097-658	
LS columns	Miltenyi Biotec		130–042-401	
Deep Well Plates	Axygen		P-DW-11-C-S	
Murine rIFN-γ	Biolegend		575306	
Falcon 50 mL high-clarity conical centrifuge tubes	Fisher Scientific		1443222	
RNAase free water	Invitrogen		AM9937	
Trypsin–EDTA (0.25%)	Gibco		25200056	
PBS	Gibco		10100223	
*Clostridium scindens*	ATCC		35704	
Brain heart infusion media	Anaerobe Systems		AS-872	
Heat-inactivated fetal bovine serum	Life Technologies		16000044	
L-Cysteine	Sigma-Aldrich		C1276	
Equipment	Supplier		Product ID	
Automatic hemocytometer	Biorad		TC-20	
Microcentrifuge	Beckman Coulter		Microfuge 16	
Tabletop Centrifuge	Beckman Coulter		Allegra V15R	
MACS magnet stand	Miltenyi Biotec		MACS Multistand	
MACS magnets	Miltenyi Biotec		MidiMACS magnets	

### Flow cytometry and statistical analyses

Spectral flow data were analyzed on web-based flow cytometry analysis software OMIQ. All data were exported into GraphPad Prism (Version 9.3.1) for statistical analysis. All figures were created in GraphPad and exported into Adobe Illustrator for arrangement (version 27.0).

## Data Availability

All data are shown and are available upon request from corresponding author Stacey L. Burgess (slburgess2@houstonmethodist.org)
